# Efficacy and Safety of COVID-19 Convalescent Plasma in Hospitalized Patients—An Open-Label Phase II Clinical Trial

**DOI:** 10.3390/life12101565

**Published:** 2022-10-09

**Authors:** Rada M. Grubovic Rastvorceva, Sedula Useini, Milena Stevanovic, Ilir Demiri, Elena Petkovic, Massimo Franchini, Daniele Focosi

**Affiliations:** 1 Institute for Transfusion Medicine of RNM, 1000 Skopje, North Macedonia; 2 Faculty of Medical Sciences, University Goce Delcev, 2000 Stip, North Macedonia; 3 University Clinic for Infectious Diseases, 1000 Skopje, North Macedonia; 4 Division of Hematology, Caro Poma Hospital, 46100 Mantua, Italy; 5 North-Western Tuscany Blood Bank, Pisa University Hospital, 56124 Pisa, Italy

**Keywords:** convalescent plasma, COVID-19, SARS-CoV-2

## Abstract

Background: COVID-19 convalescent plasma (CCP) is an important antiviral option for selected patients with COVID-19. Materials and Methods: In this open-label, phase 2, clinical trial conducted from 30 April 2020 till 10 May 2021 in the Republic of North Macedonia, we evaluated the efficacy and safety of CCP in hospitalized patients. Treatment was with a single unit of CCP having an anti-RBD IgG concentration higher than 5 AU/mL. Results: There were 189 patients that completed the study, of which 65 (34.4%) had WHO 8-point clinical progression scale score of 3 (requiring hospital care but not oxygen support), 65 (34.4%) had a score of 4 (hospitalized and requiring supplemental oxygen by mask or nasal prongs), and 59 (31.2%) had a score of 5 (hospitalized and requiring supplemental oxygen by non-invasive ventilation or high-flow oxygen). Mean age was 57 years (range 22–94), 78.5% were males, 80.4% had elevated body mass index, and 70.9% had comorbidity. Following CCP transfusion, we observed clinical improvement with increase rates in oxygenation-free days of 32.3% and 58.5% at 24 h and seven days after CCP transfusion, a decline in WHO scores, and reduced progression to severe disease (only one patient was admitted to ICU after CCP transfusion). Mortality in the entire cohort was 11.6% (22/189). We recorded 0% mortality in WHO score 3 (0/65) and in patients that received CCP transfusion in the first seven days of disease, 4.6% mortality in WHO score 4 (3/65), and 30.5% mortality in WHO score 5 (18/59). Mortality correlated with WHO score (Chi-square 19.3, *p* < 0.001) and with stay in the ICU (Chi-square 55.526, *p* ≤ 0.001). No severe adverse events were reported. Conclusions: This study showed that early administration of CCP to patients with moderate disease was a safe and potentially effective treatment for hospitalized COVID-19 patients. The trial was registered at clinicaltrials.gov (NCT04397523).

## 1. Introduction

The COVID-19 pandemic is a significant threat to public health. The disease, which has spread to 225 countries worldwide with more than 596 million confirmed cases, has caused over 6.5 million deaths [1]. Clinicians and researchers have struggled to develop effective therapeutic protocols to treat and contain the spread of COVID-19, and more than 300 drugs have been or are being investigated under clinical trials in different parts of the world [2,3,4]. Based on its historic success for a variety of infectious diseases and its overall safety [5,6,7,8,9,10,11,12,13,14,15], COVID-19 convalescent plasma (CCP) has been employed as treatment for COVID-19 since nearly the beginning of the pandemic [2,16,17,18,19,20]. CCP is readily available as soon as the first convalescent can donate plasma, affordable and deployable even in resource-poor countries [21]. The active ingredient of CCP is neutralizing antibodies (nAb) [5,7,16], which can be measured in viral neutralization assays or more practically with high-throughput surrogate assays [22]. CCP includes other components that may be beneficial, including non-neutralizing anti-Spike antibodies, antithrombin, ACE-2, albumin, and alpha-1 antitrypsin [16,23].

Randomized controlled trials (RCTs) of CCP have shown a consistent trend. To be effective, the CCP product must have sufficient titers of nAb and be used at an early stage of the infection [21]. Use of CCP with low (or no) levels of nAb or in patients who are at later stages where the disease process is driven by an overactive inflammatory response is not effective. In this regard, CCP is no different than small molecule antivirals or monoclonal antibodies, which also require early administration with sufficient drug for efficacy.

In this study, we report the results of an open-label, phase 2, clinical trial, performed in the Republic of North Macedonia (RNM) that examined the efficacy and safety of COVID-19 convalescent plasma in hospitalized patients with COVID-19.

## 2. Materials and Methods

This was an open-label, phase 2, clinical trial performed from 30 April 2020 to 10 May 2021. Recruitment of donors and collection of CCP was performed at the Institute for Transfusion Medicine of the Republic of North Macedonia (RNM) in Skopje, RNM and treatment of hospitalized COVID-19 patients was performed at University Clinic for Infectious Diseases in Skopje, RNM. One ABO-matched unit of CCP (~220 mL) was infused using standard transfusion guidelines, and recipients were followed up. CCP units were used in the same wave of COVID-19, as they were collected closely in time to when they were administered. This study was approved by the Ethical Committee of the Medical Faculty in Skopje, institute and clinic management. Informed consent was obtained from all patients. The trial was registered at clinicaltrials.gov (NCT04397523).

### 2.1. Patients

Eligibility criteria for inclusion were: Adults with laboratory confirmed SARS-CoV-2 infection (nasopharyngeal swab positive for SARS-CoV-2 by polymerase chain reaction), admitted to an acute care facility for the treatment of COVID-19 complications, patients with severe COVID-19, or patients who are at high risk of progression to severe or life-threatening disease. Informed consent provided by the patient or healthcare proxy was obtained before enrolment. Exclusion criteria were: Patients younger than 18 years, contraindication to transfusion (severe volume overload, history of anaphylaxis to blood products), patients who received immunoglobulin therapy in the past 30 days, pregnancy, and breastfeeding. None of the patients had been vaccinated for COVID-19 as vaccine was unavailable during the study period.

We collected patients’ baseline characteristics, including demographic data (e.g., age, sex, body mass index (BMI), associated drug therapy, ABO blood group and RhD factor and comorbidities (e.g., obesity, arterial hypertension, cardiovascular diseases, chronic kidney disease, chronic respiratory diseases, diabetes mellitus, hypothyroidism, etc.). We recorded the date of hospital admission, date of CCP transfusion, days between hospitalization and transfusion, days between the onset of symptoms and CCP transfusion, and dates of discharge (or death). Additionally, we recorded the WHO score (8-point clinical progression scale) at hospital admission, at 24 h, 7 days, 14 days, 21, and 28 days after CCP transfusion (where applicable), and at hospital discharge.

### 2.2. Convalescent Plasma

CCP donors were individuals who met all regular voluntary donor eligibility requirements by national regulation. Donors were male or female who have not been pregnant, or female donors who have been pregnant and tested negative for HLA antibodies, aged 18 to 60 years (if first-time donors) or 65 years (if regular donors), weighed more than >55 kg, had laboratory-confirmed previous SARS-CoV-2 infection (with RT-PCR), and were at least 21 days without symptoms from the date of two consecutive negative SARS-CoV-2 PCR test results from nasopharyngeal swab collected 24 h apart, or minimum 28 days after the last symptom or finishing of the isolation (in asymptomatic ones). Only plasma from donors whose concentration of IgG to SARS-CoV-2 exceeded 5 AU/mL was used.

Antibody testing was performed at the Institute for Immunobiology and Human Genetics in Skopje using CLIA-approved methods. Initial testing was with Snibe Maglumi 2019-nCoV IgM and 2019-nCoV IgG (qualitative) assays. Donors’ samples were retested using the Snibe Maglumi SARS-CoV-2 S-RBD IgG (quantitative) assay. An IgG concentration cutoff of >5 AU/mL was utilized to determine eligibility for CCP that would be suitable for transfusion. All potential donors were tested by RT-PCR for SARS-CoV-2 before donation, antibodies to SARS-CoV-2 and HLA (in women with previous pregnancies), full blood count, biochemistry, ABO blood group, Rh phenotyping, antibody screening, and screening for human immunodeficiency virus, hepatitis B and C, and syphilis. All CCP units used in this study were from unvaccinated donors.

The preferred method for CCP collection was plasmapheresis, which was performed with cellular separator Trima Accel (Terumo BCT) and donation of whole blood, depending on donor preference and venous access. All donors provided informed consent for donation, inclusion in the study, and for their specimen of plasma to be stored for future testing.

### 2.3. Outcomes

The primary outcome of this clinical trial was efficacy of CCP, and the secondary was safety of CCP, which were assessed through the primary and secondary endpoints.

Primary endpoints were: Duration of oxygenation and ventilation support after CCP transfusion, hospital length of stay (LOS) after CCP transfusion, intensive care unit (ICU) admission after CCP transfusion, oxygenation-free days after CCP transfusion and incidence of serious adverse events after CCP transfusion. Secondary outcomes were the type of supplemental oxygen support used in the investigated group of patients (e.g., nasal cannula, high flow nasal cannula, non-invasive ventilation, intubation and invasive mechanical ventilation, rescue ventilation) and clinical outcomes including death, critical illness, and recovery.

### 2.4. Statistical Analysis

The following statistical programs were used: STATISTICA 12.0; SPSS 20.0.

Descriptive statistic is presented as frequencies and percentages. Measures of central tendency and measures of dispersion of data are determined (mean, median, standard deviation, and interquartile range). Analytic data is presented as point estimates and 95% confidence intervals (±95% CI). P-values less than 0.05 were considered statistically significant. Percentage of structure is used, and differences are tested with the Difference test. Significance of the differences in more variables was tested with Analysis of Variance test and afterwards, with the post hoc Turkey HSD test. Significance of the differences in the two variables was tested with the Student *t*-test (t). The Pearson coefficient of correlation was used. The survival curve by Kaplan–Meier was done. Log-Rank test was used for the association of two variables, and for more variables X^2^ test was used (Pearson Chi-square). Unadjusted (crude) mortality estimates were constructed. For unadjusted mortality or case fatality rate, tabulations of the number of mortality events recorded by day 28 or until hospital discharge, divided by the total number at risk, were computed.

## 3. Results

From 30 April 2020 to 10 May 2021, 200 adult patients with COVID-19 were recruited. Of these, 189 completed the study, and 11 did not due to transfer between hospitals (8) and discharge from hospital prior to receipt of full treatment (3).

Descriptive characteristics of CCP-treated COVID-19 patients are shown in Table 1. All patients were Caucasian, and their ethnic distribution was similar to that of the population in RNM. There were 78% male patients with male/female ratio 3.6 (*p* = 0.0000).

The mean age was 57.4 ± 12.8 years (IQR 49–66, range 22–94), with more than half (54.5%) of the treated patients between the ages of 45 to 65 years and 50% older than 58. BMI distribution at enrolment was above normal range at 28.4 kg/m^2^ ± 4.4 (IQR 25.5–30.4). More than half (50.3%) were overweight, 30.1% were obese, and 19.6% had a normal body weight. Using the 8-point WHO clinical progression scale, there were 65 patients who had a score of 3 at admission (34.4%), 65 patients who had a score of 4 (34.4%), and 59 patients with a score of 5 (31.2%). According to ABO and Rh blood group distribution, there were more patients with A blood group (49.7%), which was above that of the general population in RNM. Of those, 40% were WHO score 3, 50.8% WHO score 4, and 59.3% WHO score group 5. The rate of patients with O blood group was 24.9%, which was below that of the general population in RNM. Of those, 30.7% were in WHO score 3, 23.1% in WHO score 4, and 20.3% in WHO score 5. The rate of RhD positivity was 90.5% which was above that of the general population in RNM (Table 1). Of those, 87.7% were in WHO score 3, 92.3% in WHO score 4, and 91.5% in WHO score 5 group. There were comorbidities in 70.9% of patients (Table 2): 40.3% had one, 34.3% had 2, 13.4% had 3, and 11.9% had four or more comorbidities.

There was a correlation between age and WHO scores (r = 0.16, *p* = 0.024) and between WHO scores and time since the onset of symptoms till CCP transfusion (r = 0.2341, *p* = 0.001).

The mean time between the onset of symptoms and CCP transfusion was 11.6 ± 3.3 days (10.6 ± 3.0 in WHO score 3, 12.1 ± 3.1 in WHO score 4, and 12.4 ± 3.5 in WHO score 5), while mean time from hospitalization to transfusion was 3.5 ± 2.0 days (range 1–14). There were 52.4% of patients treated with CCP from 11 to 15 days from the beginning of the disease, 28% were treated with CCP in 8 to 10 days period, 10.1% were treated with CCP more than 15 days from the beginning of the disease, and 9.5% were treated with CCP in the first seven days of the disease. There were no deaths in the group of patients that received CCP transfusion in the first seven days since the onset of symptoms.

**Figure 1:**
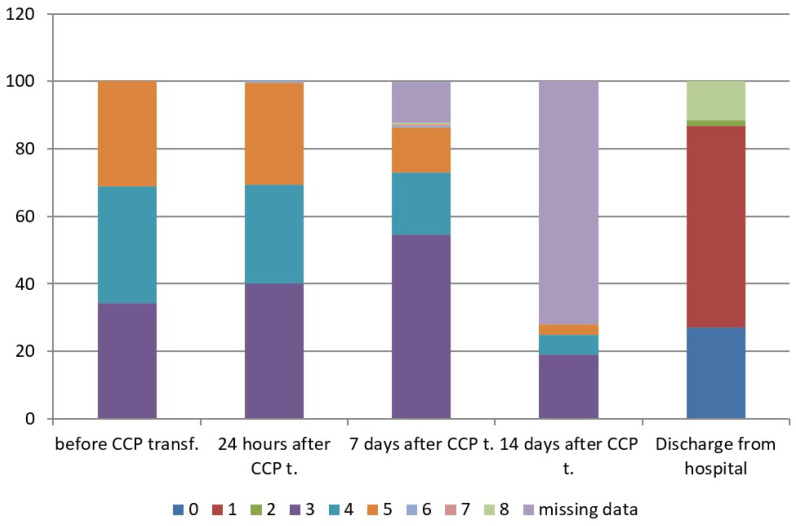
WHO scores on 8-point clinical progression scale at different time points. WHO score 0—No clinical or virological evidence of infection—uninfected; 1—No limitations in activities—ambulatory; 2—Limitation of activities—ambulatory; 3—Hospitalized, without oxygen support—moderate disease; 4—Hospitalized, oxygen by mask or nasal prongs—moderate disease; 5—Hospitalized, non-invasive ventilation (NIV) or high-flow oxygen (HFO)—severe disease; 6—Hospitalized, intubation and mechanical ventilation—severe disease; 7—Ventilation +additional organ support—pressors, RRT, ECMO—severe disease; 8—Dead.

The concomitant therapy is shown in Supplemental Table (Table S1). The mean concentration of anti-SARS-CoV-2 S-RBD IgG (AU/mL) of CCP units transfused to patients was 28.7± 23.9 AU/mL (IQR 11.3–40.8), with a range from 5.1 to 100.0 AU/mL. The mean duration of oxygenation support after CCP transfusion was 7.4 ± 5.2 days (range 1–26): It was 5.4 ± 3.5 in patients with WHO score 4 (range 1–16), and 9.4 ± 5.8 in patients with WHO score 5 (range 1–26). Hospital length of stay after CCP transfusion was 11.0 ± 5.3 days (range 3–29), in patients with WHO score 3 was 8.6 ± 2.7 days (range 3–15), in patients with WHO score 4 was 11.1 ± 4.7 days, (range 4–29) and in patients with WHO score 5 was 13.6 ± 6.7 days, (range 3–29). According to the ANOVA test, the difference between three subgroups was significant for *p* < 0.05. Afterwards, Turkey HSD test was performed and showed a difference between WHO score 3 versus WHO scores 4 and 5, and WHO score 4 versus WHO score 5 (Table 3). There was only one of 189 treated patients (0.53%), who was admitted to an intensive care unit (ICU) after CCP transfusion, while there were 39 patients already admitted to ICU before CCP transfusion. There were 65 oxygenation-free patients (34.4%) at enrolment belonging to WHO disease progression score 3 on 8-point clinical progression scale, 76 patients (40.2%) were oxygen-free 24 h after CCP transfusion, 103 patients (54.5%) were oxygen-free seven days after CCP transfusion and 167 patients (88.36%) were oxygen-free at discharge. According to the dynamic index, there was an increase rate of 32.3% of oxygenation-free days 24 h after CCP transfusion and an increase rate of 58.5% of oxygenation-free days seven days after CCP transfusion. The type of respiratory support is shown in Table 4. The clinical status of treated patients at different time points is shown in Table 5 and Figure 1, which show evident clinical improvement on the 8-point WHO clinical progression scale.

Crude mortality in the entire cohort was 11.6% (22/189). There were no deaths in patients belonging to WHO disease progression score 3 (0/65). There were no deaths in patients that received CCP transfusion in the first seven days since the onset of symptoms and in patients younger than 41 years. Crude mortality in the WHO disease progression score 4 group was 4.6% (3/65), and crude mortality in the WHO disease progression score 5 group was 30.5% (18/59), and they were all hospitalized in ICU.

**Figure 2:**
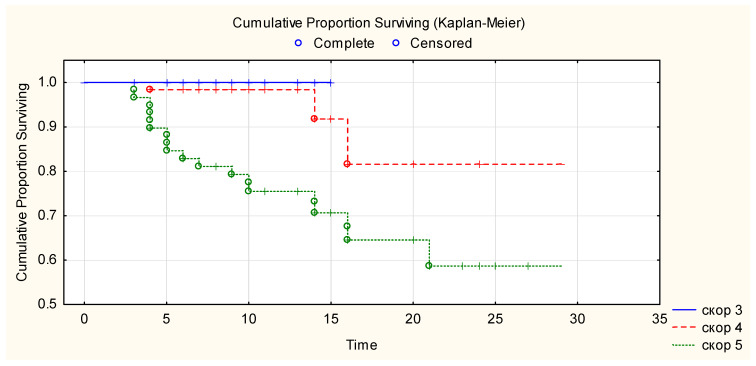
Overall survival after CCP transfusion according to their baseline WHO clinical progression scores. Figure legend: _____ (blue line)—WHO clinical progression score 3. -—- - (red line)—WHO clinical progression score 4. ……… (green line)—WHO clinical progression score 5.

Kaplan–Meier curve showed that 100% of patients with WHO progression disease score 3 survived until hospital discharge after CCP transfusion, 81% of patients with WHO progression disease score 4 survived more than 28 days after CCP transfusion, and 69% of patients with WHO progression disease score 5 survived more than 28 days after CCP transfusion (Figure 2). There is a statistically significant difference in overall survival between the three WHO disease progression score groups for *p* < 0.05 (Chi-square = 24.9, df = 2, *p* ≤ 0.00001). There was a correlation between event (mortality) versus WHO disease progression score of patients (Chi-square 19.3, *p* < 0.001) and stay in the ICU (Chi-square 55.526, *p* ≤ 0.001). There was no correlation between event (mortality) versus age, ethnicity, gender, BMI, ABO blood group, comorbidities, days from the beginning of the disease and CCP transfusion. Anti-SARS-CoV-2 RBD IgG levels in the transfused CCP units were not correlated with survival in the Log-Rank test (*p* = 0.34).

There were no serious adverse events 0 (0%). There was one patient (1/189; 0.53%) who had an urticarial skin rash during CCP transfusion, but after resolution of symptoms, he was able to tolerate the entire CCP transfusion.

## 4. Discussion

CCP is the second most frequent investigational medicinal product evaluated in COVID-19-related clinical trials, and increasing interest in this form of immunotherapy is documented by the fact that more than 140 clinical trials specifically evaluating CCP in COVID-19 have been registered to date worldwide [24,25]. This study adds to the growing body of evidence supporting the efficacy of high-titer CCP in reducing progression to severe disease [19,26,27,28] and decreasing mortality [2,20,26,29,30,31,32,33,34,35,36,37,38] in inpatients with early COVID-19 and low WHO scores. There is a biological explanation to support CCP administration early in the disease course. Viremia peaks in the first week of infection in most viral illnesses [14]. Studies have shown that viral loads are highly correlated with disease severity and progression [39,40]. Hence, patients may be at greater risk of virus-related damage and in position to gain the most benefit from antiviral therapies such as CCP during this early period [41,42,43]. Furthermore, similar to the findings of Sanz et al. [26], but in contrast to other CCP studies [30,32,34], we did not detect an association between the anti-SARS-CoV-2 titers in CCP and clinical outcomes. It is possible that this was due to the CCP units in this study having at least a minimum high level of neutralizing antibodies and that excess antibodies, once the virus has already been neutralized, would not confer additional benefit [26]. This study showed improvement in clinical findings in the investigated group of patients, with an increase of 32.3% of oxygenation-free days 24 h after CCP transfusion and an increase of 58.5% of oxygenation-free days seven days after CCP transfusion, and accordingly apparent improvement in WHO clinical progression scores. The overall mortality rate in our cohort (11.6%) is similar to that reported in recent real-life studies performed on large cohorts of hospitalized COVID-19 patients and comparable to figures reported in most previous studies on CCP [2,26,35,44,45]. There were no deaths after receiving CCP transfusion in patients with WHO score 3 (mortality 0/65; 0%) and in patients treated in the first seven days of illness. Mortality in patients belonging to WHO disease progression score 4 was 4.6% (3/65).

Franchini et al. [46] summarized 29 systematic reviews based on more than 600 overlapping reports and 53 individual primary studies (43 controlled trials, including 17 RCTs and 26 non-RCTs, and 10 uncontrolled trials-single arm studies), highlighting a mortality reduction in CCP over standard therapy when administered early and at high titer, without increased adverse reactions, despite the variability in the certainty of the evidence, mostly related to the risk of bias and inconsistency. These findings correlate with ours and with the findings of meta-analysis of Klassen et al. [47]. A similar finding, i.e., a strong inverse correlation between CCP use and mortality per hospital admission, was also observed in a publication reporting the US experience on Expanded Access Program (EAP) use of CCP in approximately 500,000 patients [2,48]. On the other hand, there have been a large number of well-executed clinical trials, such as RECOVERY, REMAPCAP, or CONCOR-1 that did not find CCP to be beneficial, albeit in advanced COVID-19 [49,50,51,52,53,54,55]. While it was not known at the time of their design, many of these studies of CCP focused disproportionately on populations (i.e., late-stage COVID-19) and interventions (low-titer CCP) that are now known to be suboptimal or ineffective for passive antibody-based therapy, whereby early administration of high-titer plasma is critical [20,49]. As an example, a subgroup analysis of the RECOVERY data for patients without the use of corticosteroids (indicative of earlier disease stage) showed a trend toward fewer deaths at 28 days in the CCP versus the control groups (19% vs. 24%, respectively). There was a similar observation in patients that did not receive respiratory support [49] and received CCP early after disease onset [26,49,52]. There are several explanations for the discrepancy between mortality rates observed in real-life studies and in RCTs, many of which failed to show a reduction in mortality and some discontinued for futility. These include the fact that CCP is not pharmaceutical but rather an artisanal product (it is produced at transfusion centers) and nAb titer and absolute content in the cumulative volume varies widely [2,56]. Additionally, differences in study design, patients’ characteristics, and disease severity could have played a role. Nevertheless, when subgroup analyses were restricted to the early use of high-titer CCP, most of the published RCT showed signals of CCP efficacy, including reductions in mortality [2,20]. Other studies have demonstrated that CCP treatment can benefit immunocompromised patients [57,58,59,60,61] as well as outpatients [62,63].

Another important finding of this study is that CCP therapy was a safe treatment with no serious adverse events among the 189 CCP units transfused. The safety of CCP has been confirmed in multiple studies [2,26,32,33,34,64,65], with the largest data set supporting a high safety profile coming from the US Expanded Access Program, which initially reported on 5000 [45], then 20,000 [66], and most recently, on 100,000 CCP recipients [2,67].

Factors associated with worse outcome in this study were increased age and stay in the intensive care unit, i.e., more advanced disease. These findings are in accordance with other studies [2,20,26,68]. The clinical characteristics of non–survivors in this study included adult males, A blood group, overweight (mostly obese), with comorbidities and hypertension as the most common, which is in line with the other studies [68,69,70,71]. Blood group A may be associated with a higher risk of SARS-CoV-2 infection along with severe disease [72,73,74]. According to a prognostic study by Park et al. [75], based on data from the COMPILE study, patients with preexisting conditions (diabetes, cardiovascular and pulmonary disease), with blood type A or AB, and at early COVID-19 stage (low baseline WHO scores) were expected to benefit most from CCP.

The main limitation of this study is that we did not have a control group of patients. Nonetheless, the result from this study demonstrated safety (no serious adverse events) and suggested efficacy, especially if given in the first seven days and to patients with mild to moderate disease (i.e., WHO scores 3 and 4 on WHO 8-score disease progression scale). Our results with CCP use in RNM are consistent with and supportive of findings in other parts of the world.

The COVID-19 pandemic is still present worldwide, and new waves of the disease are occurring, even in countries where the population has been vaccinated, due to the emergence of new variants and the decreasing efficacy of existing vaccines. Therefore, patients with a high risk of morbidity and mortality should be identified early in order to administer the best treatments available before they progress to severe disease [68]. While old CCP stocks are no longer effective at neutralizing Omicron [76], nowadays, CCP is largely available from regular donors who are likely to be also triple vaccinated. The concurrence of these two conditions creates heterologous immunity [77], which is extremely helpful at a time when the Omicron BA.2 and BA.4/BA.5 sublineages have defeated most of the anti-Spike monoclonal antibodies authorized so far [78]. While fractionated plasma products (e.g., hyperimmune globulin, monoclonal antibodies) and/or vaccination may offer durable therapeutic options, human anti–SARS-CoV-2 plasma is the only therapeutic strategy that is immediately available for the use to prevent and treat COVID-19 [16], especially in low- and middle-income countries where availability of more expensive drugs is limited. The other major advantage is versatility, with the potential for CCP to respond to emerging variants [49].

Niches for CCP treatment of COVID-19 include outpatients who are at high risk for disease progression, hospitalized patients who do not have SARS-CoV-2 antibodies detected at admission or have preexisting immunosuppression and chronically infected patients, as recently recommended by AABB and FDA guidance [79,80].

This study suggests that CCP may be as effective as other antibody-based and small-chemical antivirals [81], especially if given early in the disease course and with a high titer of antibodies. CCP can be helpful in selected patients with COVID-19, and further studies are needed to identify the subset of patients that would most benefit from it and to elucidate the optimal dose.

## 5. Conclusions

We have shown here the feasibility of a CCP program in the midst of COVID-19 pandemic, at a time when neither vaccines nor monoclonal antibodies were available in N. Macedonia. There were no deaths after receiving CCP transfusion in patients with WHO score 3 group and in those who received CCP in the first seven days of illness, and mortality of patients in WHO score 4 group was very low (3/65; 4.6%). Furthermore, CCP therapy was safe, with no serious adverse events encountered among the 189 CCP units transfused.

In conclusion, based on our experience, the use of CCP in hospitalized COVID-19 patients was characterized by high safety and efficacy, when administered early in the disease course with high titer of antibodies, particularly in patients with moderate disease.

## Figures and Tables

**Table 1:** Demographic characteristics of randomized COVID-19 patients.

Patients	Number	%	Number	%	Number	%	Number	%
WHO Disease Progression Scores—before CCP transfusion	3		4		5		Whole investigated group (all scores)	
Patients	65	34.4	65	34.4	59	31.2	189	100
Ethnicity								
Others	4	6.2	6	9.2	4	6.8	14	7.4
Macedonians	45	69.2	53	81.5	41	69.5	139	73.5
Albanians	16	24.6	6	9.2	14	23.7	36	19.1
Gender								
Male	52	80.0	48	73.8	48	81.4	148	78.3
Female	13	20.0	17	26.2	11	18.6	41	21.7
BMI								
18.5–24.9 (normal)	14	21.6	11	16.9	12	20.3	37	19.6
25–29.9 (overweight)	37	56.9	34	52.3	24	40.7	95	50.3
≥30.0 (obese)	14	21.5	20	30.8	23	39.0	57	30.1
Blood Group								
B	14	21.5	11	16.9	7	11.9	32	16.9
A	26	40.0	33	50.8	35	59.3	94	49.7
O	20	30.7	15	23.1	12	20.3	47	24.9
AB	5	7.7	6	9.2	5	8.5	16	8.5
Comorbidities								
No	28	43.1	16	24.6	11	18.6	55	29.1
Yes	37	56.9	49	75.4	48	81.4	134	70.9
RhD factor								
Negative	8	12.3	5	7.7	5	8.5	18	9.5
Positive	57	87.7	60	92.3	54	91.5	171	90.5
Age								
<35	7	10.8	2	3.1	2	3.4	11	5.8
36–45	6	9.2	8	12.3	9	15.3	23	12.2
46–55	20	30.8	15	23.1	12	20.3	47	24.9
56–65	22	33.8	19	29.2	15	25.4	56	29.6
66–75	7	10.8	18	27.7	16	27.1	41	21.7
>75	3	4.6	3	4.6	5	8.5	11	5.8

**Table 2:** Distribution of comorbidities in recruited patients.

Comorbidities	Number of Patients	N = 134%
HTA (hypertension)	74	55.2
DM (diabetes mellitus)	34	25.4
Obesity	53	39.6
CVD (cardiovascular diseases) (other than HTA), such as AFF, tachycardia, aneurism, stenting	23	17.2
Hypothyroidism	8	6.0
CRD (chronic respiratory disease)—HOBB, asthma, etc.	12	9.0
St. post carcinoma	3	2.2
BHP (benign hyperplasio of prostate)	3	2.2
CVD (cerebrovascular disease—St. post CVI and others)	3	2.2
Psychiatric disease (anxiodepresive syndrome, depressive syndrome, schizophrenia)	7	5.2
Psoriasis (psoriatic rheumatoid arthritis)	3	2.2
Thrombocytopenia	3	2.2
CRI (chronic renal insufficiency)	2	1.5
GIT (gastrointestinal diseases—St. post ulcus bulbi duodeni, cholecystectomy, Morbus Gilber)	5	2.6
Neurological diseases (Parkinson, epilepsy)	3	2.2
Hepatitis B	2	1.5
Gout	3	2.2
Macrocytic anemia	1	0.7

**Table 3:** Study outcomes.

	Entire Cohort	WHO Score 3	WHO Score 4	WHO Score 5	ANOVA Differences between Groups
Duration of oxygenation support after CCP transfusion (days) (mean ± standard deviation)	7.4 ± 5.1	not oxygenated	5.4 ± 3.5	9.4 ± 5.8	n.a.
Total length of hospital stay after CCP transfusion (days) (mean ± standard deviation)	11.0 ± 5.2	8.6 ± 2.7	11.1 ± 4.7	13.6 ± 6.7	*p* < 0.05
ICU admissions	1	0	0	1	n.a.

**Table 4:** Type of respiratory support.

	Number	%
Overall number of participants analyzed	189	100
Nasal cannula, oxygen mask before CCP transfusion	65	34.4
Nasal cannula, oxygen mask 24 h after CCP transfusion	56	29.6
Nasal cannula, oxygen mask 7 days after CCP transfusion	35	18.5
Non-invasive ventilation, high flow nasal cannula before CCP transfusion	59	31.2
Non-invasive ventilation, high flow nasal cannula 24 h after CCP transfusion	56	29.6
Non-invasive ventilation, high flow nasal cannula 7 days after CCP transfusion	25	13.2
Intubation or invasive mechanical ventilation before CCP transfusion	0	0
Intubation or invasive mechanical ventilation 24 h after CCP transfusion	1	0.5
Intubation or invasive mechanical ventilation 7 days after CCP transfusion	1	0.5
Rescue ventilation before CCP transfusion	0	0
Rescue ventilation 24 h after CCP transfusion	0	0
Rescue ventilation 7 days after CCP transfusion	1	0.5

**Table 5:** WHO scores on 8-point clinical progression scale at different time points.

WHO Disease Progression Scores before CCP Transfusion	Number	%
WHO score 3—Hospitalized, without oxygen support—moderate disease	65	34.4
WHO score 4—Hospitalized, oxygen by mask or nasal prongs—moderate disease	65	34.4
WHO score 5—Hospitalized, non-invasive ventilation (NIV) or high-flow oxygen (HFO)—severe disease	59	31.2
WHO scores 24 h after CCP transfusion		
WHO score 3—Hospitalized, without oxygen support—moderate disease	76	40.2
WHO score 4—Hospitalized, oxygen by mask or nasal prongs—moderate disease	56	29.1
WHO score 5—Hospitalized, non-invasive ventilation (NIV) or high-flow oxygen (HFO)—severe disease	56	29.1
WHO score 6—Hospitalized, intubation and mechanical ventilation—severe disease	1	0.5
WHO scores 7 days after CCP transfusion		
WHO score 3—Hospitalized, without oxygen support—moderate disease	103	54.5
WHO score 4—Hospitalized, oxygen by mask or nasal prongs—moderate disease	35	18.5
WHO score 5—Hospitalized, non-invasive ventilation (NIV) or high-flow oxygen (HFO)—severe disease	25	13.2
WHO score 6—Hospitalized, intubation and mechanical ventilation—severe disease	1	0.5
WHO score 7—Ventilation + additional organ support—pressors, RRT, ECMO—severe disease	1	0.5
WHO score 8—Dead	1	0.5
WHO scores 14 days after CCP transfusion		
WHO score 3—Hospitalized, without oxygen support—moderate disease	36	19.0
WHO score 4—Hospitalized, oxygen by mask or nasal prongs—moderate disease	11	5.8
WHO score 5—Hospitalized, non-invasive ventilation (NIV) or high-flow oxygen (HFO)—severe disease	6	3.2
WHO scores 21 days after CCP transfusion		
WHO score 3—Hospitalized, without oxygen support—moderate disease	7	3.7
WHO score 5—Hospitalized, non-invasive ventilation (NIV) or high-flow oxygen (HFO)—severe disease	1	0.5
WHO scores 28 days after CCP transfusion		
	0	
WHO scores at hospital discharge		
WHO score 0—no clinical or virological evidence of infection—uninfected	51	27.0
WHO score 1—no limitations in activities—ambulatory	113	59.8
WHO score 2—limitation of activities—ambulatory	3	1.6
WHO score 8—dead	22	11.6

## Data Availability

All relevant data are summarized in the manuscript. Additional anonymous data are available on request from the corresponding author with justified explanation why is needed.

## Supplementary Materials

The following supporting information can be downloaded at: https://www.mdpi.com/article/10.3390/life12101565/s1, Table S1: Concomitant therapy.

## Abbreviation

CCP: COVID-19 convalescent plasma.
