# Supplementary material for: Efficacy and Safety of COVID-19 Convalescent Plasma in Hospitalized Patients—An Open-Label Phase II Clinical Trial
Source: Life (Basel). 2022 Oct 9;12(10):1565. doi: 10.3390/life12101565 (PMC9605182; doi:10.3390/life12101565)
Supplement: Supplementary file 1 [file life-12-01565-s001.zip › life-1930505-supplementary.pdf]

Table S1. Concomitant therapy

Concomitant therapy	Number	%
Antibiotics	189	100.0
Anticoagulants	186	98.4
Corticosteroids	155	82.0
Gastroprotective therapy	188	99.5
Hepatoprotective therapy	20	10.6
Antiviral drug - Remdesivir	21	11.1
Antihypertensive therapy	11	5.8
Other therapy for cardiovascular diseases	24	12.7
Diuretics	33	17.5
Bronchodilatator drugs	16	8.5
Symptomatic therapy	84	44.4
Antiplatelet therapy	9	4.8
Psychiatric drugs	1	0.5
Antidiabetic drugs	9	4.8
Antimycotic drugs	3	1.6
Dopamine	1	0.5
Tolicizumab	1	0.5
Noradrenalin	2	1.1
Lopinavir	2	1.1
Adrenalin	1	0.5
Oxygen support	124	65.6
